# Dr. J. M. Da Costa

**Published:** 1900-10

**Authors:** 


### OBITUARY NOTES.

Dr. J. M. Da Costa, of Philadelphia, Pa., died suddenly at his country home, September 12, 1900, aged 67 years. He was a distinguished teacher and author, having published many books, monographs and brochures. His most enduring work is his classic, "Medical Diagnosis," that appeared in 1864, and has gone through many editions.—Dr. Hunter McGuire, of Richmond, Va., died at his country home near that city, September 19, 1900, aged 65 years. Dr. McGuire served with distinction as a surgeon in the Confederate Army, and for some years after the war he occupied a prominent place in the South as a surgeon.—Dr. Alfred Stillé, of Philadelphia, died at his home September 24, 1900, aged 87 years. He was at one time a teacher and author of note, and had been president of the American Medical Association.—Dr. Preston B. Scott, of Louisville, Ky., president of the Confederate Army and Navy Surgeons' Association, died September 24, 1900, aged 68 years.—Dr. Franklin Burritt, of Fredonia, N. Y., at one time a member of the State Board of Charities and a manager of the Buffalo State Hospital, died at his home, September 23, 1900, aged 73 years.—Dr. Lewis A. Sayre, of New York, died at his home, September 21, 1900, aged 81 years. Dr. Sayre had been a surgeon of distinction and was famous in the treatment of deformities. His plaster jacket in diseases of the spine was a valuable contribution to the treatment of spinal affections.

